# A Diplodocid Sauropod Survivor from the Early Cretaceous of South America

**DOI:** 10.1371/journal.pone.0097128

**Published:** 2014-05-14

**Authors:** Pablo A. Gallina, Sebastián Apesteguía, Alejandro Haluza, Juan I. Canale

**Affiliations:** 1 CONICET, Buenos Aires, Argentina; 2 Fundación de Historia Natural Félix de Azara, Universidad Maimónides, Buenos Aires, Argentina; 3 Museo Paleontológico Ernesto Bachmann, Villa El Chocón, Neuquén, Argentina; Raymond M. Alf Museum of Paleontology, United States of America

## Abstract

Diplodocids are by far the most emblematic sauropod dinosaurs. They are part of Diplodocoidea, a vast clade whose other members are well-known from Jurassic and Cretaceous strata in Africa, Europe, North and South America. However, Diplodocids were never certainly recognized from the Cretaceous or in any other southern land mass besides Africa. Here we report a new sauropod, *Leikupal laticauda* gen. et sp. nov., from the early Lower Cretaceous (Bajada Colorada Formation) of Neuquén Province, Patagonia, Argentina. This taxon differs from any other sauropod by the presence of anterior caudal transverse process extremely developed with lateroventral expansions reinforced by robust dorsal and ventral bars, very robust centroprezygapophyseal lamina in anterior caudal vertebra and paired pneumatic fossae on the postzygapophyses in anterior-most caudal vertebra. The phylogenetic analyses support its position not only within Diplodocidae but also as a member of Diplodocinae, clustering together with the African form *Tornieria*, pushing the origin of Diplodocoidea to the Middle Jurassic or even earlier. The new discovery represents the first record of a diplodocid for South America and the stratigraphically youngest record of this clade anywhere.

## Introduction

As *Tyrannosaurus rex* is for theropods, *Diplodocus* [1]–[4] and *Apatosaurus* [5]–[8] are by far the most emblematic sauropod dinosaurs. They are known from nearly complete skeletons found during the late 19^th^ and early 20^th^ centuries in North America. Both genera can be easily recognized, with their large bodies capped by extremely long necks and tails. Both genera bear elongated and biconvex distal caudal vertebrae, and anteroposteriorly extended skulls with narrow teeth restricted to the distal snout [9]. These taxa are members of the Diplodocidae, a family recorded in Late Jurassic strata from North America, Europe, and Africa [10]. Diplodocids are part of the Diplodocoidea, a vast clade whose other members (e.g., Rebbachisauridae and Dicraeosauridae) are well-known from Jurassic and Cretaceous strata in Africa, Europe, North and South America. Diplodocids were not recorded from any other southern land mass besides Africa, although their occurrence in South America was potentially expected [10], [11].

Until now, the lack of reliable evidence for the survival of Diplodocidae after the Jurassic/Cretaceous boundary [12] led authors to propose an extinction event for the group at that time [13], [14]. However, this absence has also been potentially attributed to taphonomic or sampling biases driven by worldwide sea level changes registered for the early Lower Cretaceous [15]–[17].

Here, we report a new sauropod dinosaur from the early Lower Cretaceous of Neuquén Province, Patagonia, Argentina. This discovery represents the first record of a diplodocid for South America and the youngest record of Diplodocidae in the world. Furthermore, the presence of a diplodocid in Argentina augments the list of sauropod clades for this country, now including not only basal eusauropods but also all neosauropod clades, both basal and derived forms of Macronaria, as well as both basal and derived forms of Diplodocoidea, thus turning the area into an extremely rich portrait of sauropod evolution [18]–[20].

## Methods

### Nomenclatural Acts

The electronic edition of this article conforms to the requirements of the amended International Code of Zoological Nomenclature, and hence the new names contained herein are available under that Code from the electronic edition of this article. This published work and the nomenclatural acts it contains have been registered in ZooBank, the online registration system for the ICZN. The ZooBank LSIDs (Life Science Identifiers) can be resolved and the associated information viewed through any standard web browser by appending the LSID to the prefix "http://zoobank.org/". The LSID for this publication is: urn:lsid:zoobank.org:pub: EDBAE559-EDE4-4262-A972-95CFC5C6B2DD. The electronic edition of this work was published in a journal with an ISSN, and has been archived and is available from the following digital repositories: PubMed Central, LOCKSS.

### Phylogenetic analysis

The data matrices used are based on previously published neosauropod phylogenies which focused on Diplodocoidea [21] and Diplodocidae [22] relationships, with the addition of this new taxon. Two vertebral characters were added to both datasets, resulting in a data matrix of 191 characters and 27 taxa (Dataset S1), and 236 characters and 14 taxa (Dataset S2), respectively (Text S1). The datasets were analyzed using TNT v.1.1 [23], with a heuristic search of 1,000 replicates of Wagner trees followed by TBR (tree bisection-reconnection) branch swapping. Bremer support [24] and bootstrap resampling were used to evaluate the robustness of the nodes of the most parsimonious trees in both analyses.

### Specimen and repository information

The fossil quarry comprises an approximately 30 m^2^ area (Figure S1) which includes numerous intermixed specimens of different dinosaur clades organized in multiple fossiliferous levels. Only bones from two different sauropod groups are present in the fossiliferous site: Diplodocidae and Dicraeosauridae. Though the remains from both clades are mostly disarticulated and mixed, only those bones with undoubted diplodocid features are described here. This was made not only based on the general anatomy but also after a comparison between skeletal elements from equivalent anatomical positions in both clades. Doubtful or ambiguous materials were not included in this description. The specimens are housed at the Museo Municipal “Ernesto Bachmann”, Villa El Chocón, Neuquén province, under the collection number MMCH-Pv 63-1/8. All necessary permits were obtained for the described study, which complied with all relevant regulations. Secretaría Cultura Neuquén provided research permits for fieldwork and laboratory studies (Exp. 4040-002693/2009, Dict. 63/09).

## Results

### Systematic Paleontology

Dinosauria Owen, 1842

Saurischia Seeley, 1888

Sauropoda Marsh, 1878

Diplodocoidea Marsh, 1884

Flagellicaudata Harris & Dodson, 2004

Diplodocidae Marsh, 1884

Diplodocinae Marsh, 1884; Janensch, 1929

Leinkupal laticauda gen. et sp. nov.

urn:lsid:zoobank.org:act:C0C69F2B-D85C-4E20-BB8D-FE81C1CCAD7D

#### Etymology

From *lein*, vanishing, and *kupal*, family. These are Mapudungun words, the language of the Mapuche Native American nation that inhabits northwestern Patagonia. The terms refer to the record of the last known representative of the family Diplodocidae. Meanwhile, *lati*, from *latus*, wide, and *cauda*, tail, in Latin words, refer to the broad tail evidenced by the lateral extension of the transverse processes in proximal caudal vertebrae.

#### Holotype

MMCH-Pv 63-1 (Museo Municipal “Ernesto Bachmann,” Villa El Chocón, Neuquén,), includes one anterior caudal vertebra (Caudal 7, see Description and comparisons below).

#### Paratype

Two anterior cervical vertebrae (MMCH-Pv 63-2/3), one posterior cervical vertebra (MMCH-Pv 63-4), one anterior dorsal vertebra (MMCH-Pv 63-5), one anterior caudal vertebra (MMCH-Pv 63-6) and two mid-caudal vertebrae (MMCH-Pv 63-7/8).

#### Horizon and locality

The remains were found in outcrops of the Bajada Colorada Formation (Neuquén Basin), at its type locality 40 km south of Picún Leufú town on the national route 237, in southeastern Neuquén Province, Patagonia, Argentina. This unit is composed of red and greenish-brown, fine to coarse grained conglomerates and sandstones with well developed bands of light brown siltstones and reddish claystones. The unit is mostly dominated by a fluvial regime, and the paleoenviroment resembles braided river systems where well-preserved channels with cross stratification and paleosols are present [25], [26]. The fossiliferous locality also provided abundant remains of a dicraeosaurid sauropod and a diverse record of theropods, represented mainly by teeth and some bones, corresponding to basal tetanurans, possible deinonychosaurians, and possible abelisauroids.

#### Age

Seismological, surface geological, and biostratigraphical studies confirm an Early Cretaceous age (late Berriasian–Valanginian) of this formation [25], [27]. The Bajada Colorada Formation is considered a continental red bed unit, which conformably overlies the marine Picún Leufú Formation (Tithonian–early Berriasian) and is unconformably covered by the marine Agrio Formation (late Valanginian– late Hauterivian) [18], [25].

#### Diagnosis

A derived diplodocid diagnosed by the following autapomorphic traits: anterior caudal transverse process extremely developed (about equal or wider to centrum width) with lateroventral expansions reinforced by robust dorsal and ventral bars; very robust centroprezygapophyseal lamina in anterior caudal vertebra; paired pneumatic fossae located on the base of the postzygapophysis, opposite to the articular side, in anterior-most caudal vertebra.

### Description and comparisons

#### Cervical and dorsal vertebrae

**Figure 1:**
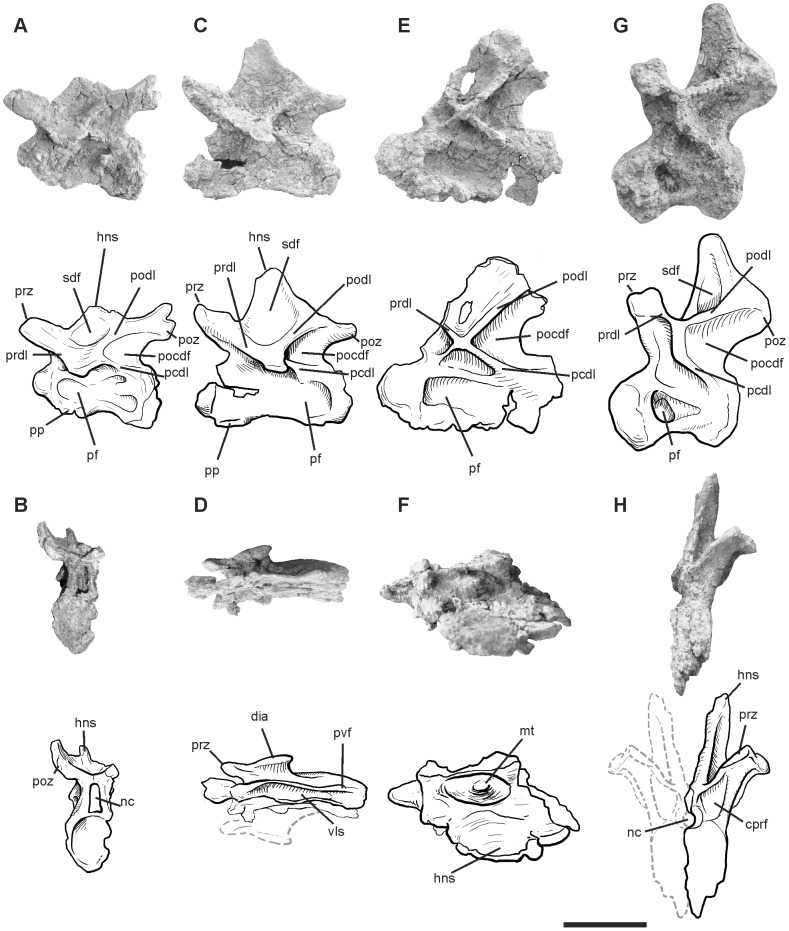
Photographs and half-tone drawings of the cervical and dorsal vertebrae of *Leinkupal laticauda*, gen. n. sp. n. (MMCH-Pv 63). Cervical 6? in (A) lateral and (B) posterior views. Cervical 8? in (C) lateral and (D) ventral views. Cervical 11? in (E) lateral and (F) dorsal views (reversed). Dorsal 2? in (G) lateral and (H) anterior views (reversed). Abbreviations: cprf, centroprezygapophyseal fossa; dia, diapophysis; hns, hemi neural spine; mt, median tubercle; nc, neural canal; pcdl, posterior centrodiapophyseal lamina; pf, pneumatic fossa; pocdf, postzygapophyseal centrodiapophyseal fossa; podl, postzygodiapophyseal lamina; poz, postzygapophysis; pp, parapophyses; prdl, prezygodiapophyseal lamina; prz, prezygapophysis; pvf, posteroventral flanges; sdf, spinodiapophyseal fossa. Scale bar equals 10 cm.

Although incomplete, three cervical vertebrae and one dorsal vertebra are preserved (Figure 1). All of them lack partially or totally one of their lateral faces. Proportionally, these vertebrae resemble the sixth, eighth, and eleventh cervical vertebrae of *Apatosaurus* [8] and the second dorsal vertebra of *Diplodocus* [3]. The anterior-most vertebra (C6) is nearly complete, except for the tip of the neural spine and the partially damaged right lateral face. The other three vertebrae (C8, C11 and D2) are only partly preserved.

The centrum length varies from two times to three times the cotyle height in the first three elements, decreasing to one and a half in the anterior dorsal (Table S1). All centra are slightly compressed in the lateromedial direction, and they show well-developed lateral pneumatic fossae on their lateral faces; however, poor preservation obscures the complexity of the pneumatization. Only in C6 does the single anterior pneumatic fossae bifurcate posteriorly, differing from the more complex configuration present in *Diplodocus* [3]. Conversely, the other cervical vertebrae preserve a unique lateral fossa that is widely extended in C8 and C11. In D2, the lateral pneumatic fossa is restricted to the anterior half of the centrum. Ventrally, a longitudinal sulcus is well-developed in the preserved C6 and C8 centra, as in all diplodocids [10].

The neural arches are taller than the centra in all preserved elements, although this ratio increases along the sequence. In C6, the neural canal is markedly high and resembles a Romanesque arch with a semicircular dorsal edge. In contrast, the other cervical vertebrae show circular neural canals, also present in *Diplodocus* [3]. The spinoprezygapophyseal lamina (sprl) in C6 seems to be interrupted at the base of the prezygapophysis, as in *Kaatedocus* [22] and other diplodocines. An incipient bifurcated neural spine occurs in C6, which is clearly defined in the rest of the cervical vertebrae. Although slightly developed on C6, a well-marked median tubercle occurs on the dorsal face between the bifid neural spine of C11, as in *Apatosaurus* [8] and *Diplodocus* [3].

Laterally, prezygodiapophyseal (prdl) and postzygodiapophyseal (podl) laminae, as well as the spinodiapophyseal fossa (sdf) above them, are well developed in the preserved vertebrae. Posterior centrodiapophyseal laminae (pcdl) are also well developed and ventrally frame the triangular and deep postzygapophyseal centrodiapophyseal fossa (pocdf). On the other hand, the anterior centrodiapophyseal lamina (acdl) is reduced in all elements.

#### Caudal vertebrae

**Figure 2:**
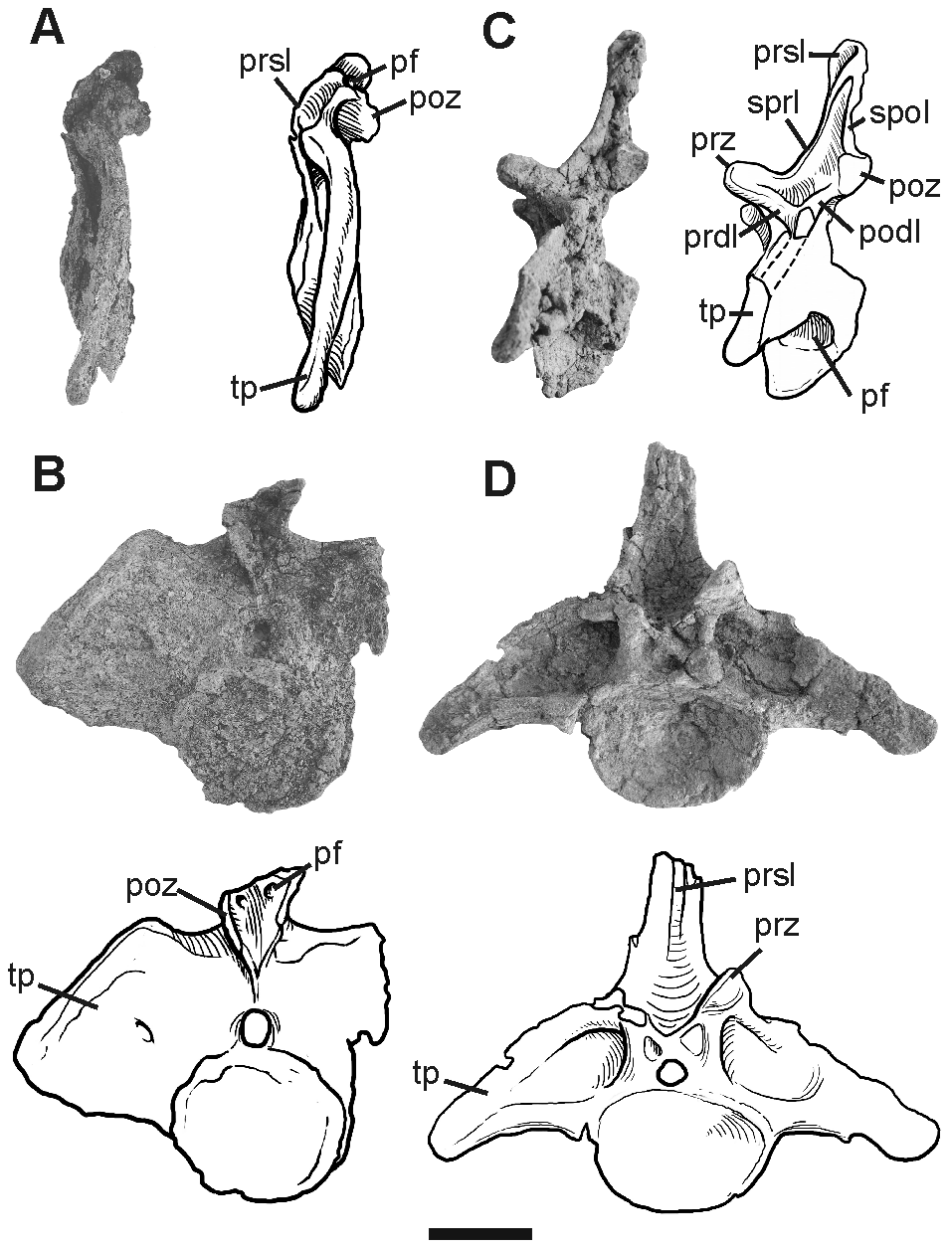
Photographs and half-tone drawings of the anterior caudal vertebrae of *Leinkupal laticauda*, gen. n. sp. n. (MMCH-Pv 63). Caudal 1-2? in (A) lateral and (B) posterior views. Caudal 7? in (C) lateral and (D) anterior views (reversed). Abbreviations: pf, pneumatic fossa; podl, postzygodiapophyseal lamina; poz, postzygapophysis; prdl, prezygodiapophyseal lamina; prsl, prespinal lamina; prz, prezygapophysis; sprl, spinoprezygapophyseal lamina; spol, spinopostzygapophyseal lamina; tp, transverse process. Scale bar equals 10 cm.

**Figure 3:**
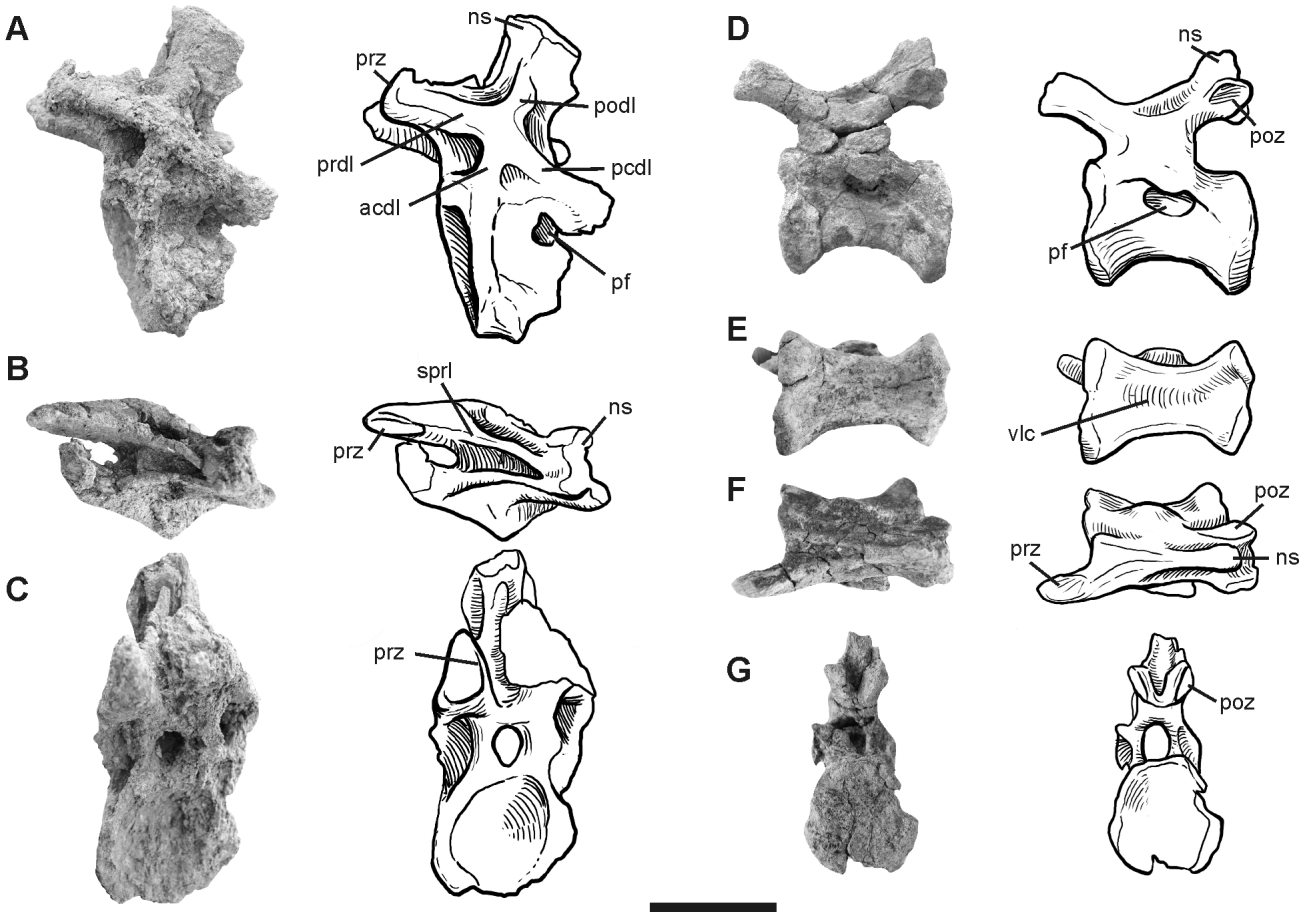
Photographs and half-tone drawings of the mid caudal vertebrae of *Leinkupal laticauda*, gen. n. sp. n. (MMCH-Pv 63). Caudal 12? in (A) lateral, (B) dorsal and (C) anterior views (reversed). Caudal 20? in (D) lateral, (E) ventral, (F) dorsal and (G) posterior views. Abbreviations: acdl, anterior centrodiapophyseal lamina; ns, neural spine; pcdl, posterior centrodiapophyseal lamina; pf, pneumatic fossa; podl, postzygodiapophyseal lamina; poz, postzygapophysis; prdl, prezygodiapophyseal lamina; prz, prezygapophysis; sprl, spinoprezygapophyseal lamina; vlc, ventral longitudinal concavity. Scale bar equals 10 cm.

Two anterior (Figure 2) and two mid-caudal vertebrae (Figure 3) are preserved. Based on the great size of the centrum and the dorsoventral extension of the transverse processes, the anterior-most element may correspond to the first or the second caudal vertebra (Ca1-2). The following element is assigned as the seventh caudal (Ca7), which preserves laterally elongated transverse processes with ends pointing ventrally as observed in other diplodocids [3], [28], [11]. The anterior mid-caudal, tentatively assigned as the twelfth (Ca12) is incomplete, laterally compressed, but conserves well-defined diapophyseal laminae and fossae. Conversely, the posterior mid-caudal (tentatively assigned as Ca20) is nearly complete, with both centrum and neural arch well preserved, excepted for the right prezygapophysis, which is missing.

The centra are somewhat procoelous, anteroposteriorly short in Ca1-2 and Ca7, and more extended in Ca20. By contrast, Ca1-2, Ca12 and Ca20 centra have subcircular perimeters nearly equally tall as wide, Ca7 is dorsoventrally low. The ventral faces of anterior elements are smooth, without longitudinal keels. In Ca20 a broad, ventral longitudinal concavity is developed as in other diplodocids, such as *Supersaurus* [29], [30], *Tornieria*, *Diplodocus* and *Barosaurus* [31]. Well-developed ovoidal lateral pneumatic fossae persist in Ca20. At present, the extension of lateral pneumatic fossae into middle caudal centra is potentially restricted to diplodocids.

The neural arches are nearly complete in the preserved elements, except for the neural spines in Ca1-2 and the summit of the neural spine in Ca7. The transverse processes are laminar and wing-like in Ca1-2, becoming a reinforced structure and lateroventrally extended (about equal to centrum width) in Ca7. The extreme development of the transverse process in Ca7 of *Leinkupal* is considered an autapomorphy. Prezygapophyses are ovoidal and flat, reduced in Ca1-2, and more conspicuous in the other caudal vertebrae. The prespinal lamina is restricted to the dorsal part of preserved neural spines in Ca1-2 and Ca7. Very robust centroprezygapophyseal laminae are developed in Ca1-2 and Ca7, a condition not present in other sauropods. A paired pneumatic fossae located on the base of the postzygapophysis in Ca 1-2 is also recognized as an autapomorphy of this taxon. Thick and well-developed diapophyseal laminae occur in Ca7 and Ca12, as in most diplodocids [10], [22]. The spinoprezygapophyseal lamina (sprl) and the spinopostzygapophyseal lamina contact each other at the mid-length of the neural spine in Ca7, as in most diplodocids [10].

### Phylogenetic analysis

**Figure 4:**
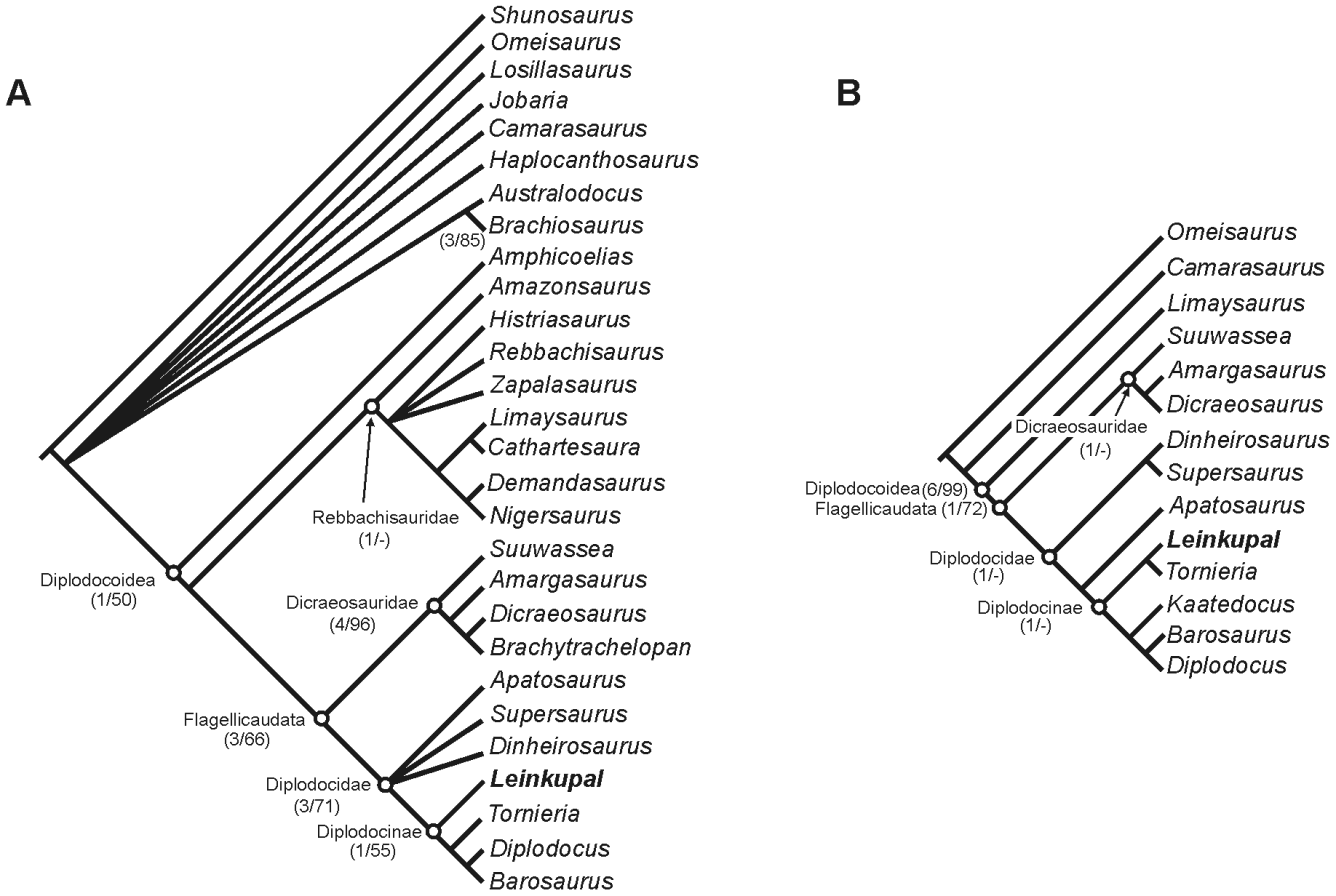
Phylogenetic position of *Leinkupal laticauda*, gen. n. sp. n. (A) Strict consensus tree recovered after the inclusion of *Leinkupal laticauda* in a published data matrix focused on Diplodocoidea relationships [21]. (B) Most parsimonious tree recovered after the inclusion of *Leinkupal laticauda* in another published data matrix focused on Diplodocidae relationships [22]. Support values (Bremer/Bootstrap) of principal nodes are in brackets.

Two different analyses were carried out in order to establish the phylogenetic position of *Leinkupal laticauda* among diplodocoids. In the first analysis we included the taxon in a published Diplodocoidea data matrix [21]. It resulted in 156 equally most parsimonious trees of 344 steps (CI = 0.605, RI = 0.770). A strict consensus tree (Figure 4A) recovers *Leinkupal laticauda* deeply nested within Diplodocidae, as the sister taxon of the African *Tornieria*, and the North American *Barosaurus* and *Diplodocus*.

After these results relating *Leinkupal* to diplodocids, we included *Leinkupal* in a different published data matrix [22], performed in order to resolve the relationships within Diplodocidae. In this second analysis, one most parsimonious tree of 371 steps (CI = 0.674, RI = 0.615) was recovered (Figure 4B). The tree recovers *Leinkupal laticauda* as the sister taxon to *Tornieria* and as a member of Diplodocinae (taxa more closely related to *Diplodocus* than to *Apatosaurus* [32]). A list of synapomorphies supporting the principal nodes of the trees from both analyses is given in Text S2. Bremer and bootstrap support values are shown in Figure 4. Derived characters shared by the new taxon with other diplodocines include: bifurcation of neural spines present on middle cervicals (97.2), absence of paired pneumatic fossae on ventral surface in anterior cervicals (102.0), spinoprezygapophyseal lamina reduced to ridge or totally interrupted in the middle (at base of prezygapohysis) in anterior and mid-cervicals (103.1), mid- and posterior cervical centra with longitudinal flanges in the lateroventral edge on the posterior part of the centrum (113.1), large coels in anterior caudal centra (173.1), and a ventral longitudinal hollow in anterior and mid-caudal centra (185.1).

## Discussion

The Diplodocidae, originally known from North America, were later discovered in Late Jurassic and possibly earliest Cretaceous sites from Europe (i.e., Spain, Portugal and Georgia) [21], [33]. Tentative reports from Asia [34] were later suggested to be non-diplodocid [12]. For Gondwanan landmasses, only the Upper Jurassic of Tendaguru beds of Tanzania produced remains of this family, known today as *Tornieria africana* [35]. That taxon represented up to now the only known Gondwanan diplodocid, but Remes [11] in his revision of the African material declared that it was expected to find other diplodocids in the Upper Jurassic of both South America and Africa. Surprisingly, the materials of *Leinkupal laticauda* appeared not in Jurassic but in Early Cretaceous strata from South America, demonstrating an extended survival of the lineage as compared to previous knowledge.

*Leinkupal* is the second confirmed diplodocid for Gondwana and the first for South America. Although the other clades of Diplodocoidea were already recorded in South America (i.e., Rebbachisauridae, Dicraeosauridae), the occurrence of Diplodocidae in South America helps to depict a more complex paleobiogeographical panorama. Furthermore, a diplodocid from the Early Cretaceous (Late Berriasian–Valanginian) provides new insights into the survival of this group relative to the other forms of the clade, which mostly come from Upper Jurassic (Kimmeridgian–Tithonian) beds of Africa and North America.

The history of the three diplodocoid groups alternatively depicts a more complex or a very simple panorama. Carballido et al. [36] considered an origin of the clade by Oxfordian times. However, several paleogeographic reconstructions suggest that seafloor spreading around the Americas had initiated by early Bajocian times (175 Ma) [37], with abundant oceanic floor both in the Mexico Gulf and western Tethys. Furthermore, by the early Late Jurassic, a global transgression flooded most coasts, helping to isolate faunas [38]. In this context, it is very likely that, as previously expressed [11], North American (pre-Morrison) and Eastern African (pre-Tendaguru) histories were already vicariant by Callovian times. Thus, the three diplodocoid groups should have originated by Bajocian times.

Within Diplodocoidea, we know that rebbachisaurids were distributed after a long ghost lineage, in the Lower Cretaceous of western Europe [39]–[41], Africa [42], [43], and South America [44]–[49], excluding up until now Asia and North America. The group was considered [36] as originating by Oxfordian times, or as discussed above, by Bajocian or Bathonian times. That contribution, thorough a DEC analysis, retrieves Rebbachisauridae as South American in origin, despite the Hauterivian age of *Histriasaurus* and the Barremian scapula from the Isle of Wight [41]. Furthermore, several authors [50]–[53] proposed that the absence of rebbachisaurids in the earliest strata of the Cretaceous in South America might be the result of regional extinction, taxonomic misidentification, the incompleteness of the fossil record, or some combination of these factors. In this context, rebbachisaurid teeth in the La Amarga Formation [48] support a long Cretaceous continuity, as expected previously [36]. Rebbachisaurids attained a wide distribution, prior to the complete fragmentation of Pangea, with a later and perhaps rapid expansion to Africa, and from it to Europe during Barremian-Aptian times, probably across Apulia [52], [54]. Differing from flagellicaudatans, rebbachisaurids reached a late diversity peak between Aptian and Turonian [55], [44], [46], [56], when they became extinct along with carcharodontosaurid theropods [57].

Dicraeosaurids have a restricted record, including only the Upper Jurassic of North America [58], Africa [59], and South America [60], where they survived until the Early Cretaceous [61], [62]. The presence of the basal *Suuwassea* in North America and its more related forms in Gondwana indicates an early origin, no later than Bathonian, for the group. The clade appeared prior to the separation of Laurasia and Gondwana, and the representatives developed their peculiarities in the context of their vicariant evolution. On the other hand, the sister group relationships between the Upper Jurassic African *Dicraeosaurus*, living in the north with respect to the Central Gondwanan Desert, and *Brachytrachelopan*, living in the south with respect to the desert, suggest that these forms had no problems in crossing this extensive region.

Diplodocids are much better-known, mainly represented up to now by an essentially Kimmeridgian-Tithonian group with no survival in the Cretaceous after a global extinction proposed for the Jurassic/Cretaceous boundary, along with several other sauropod lineages [13], [14]. However, a few remains were used as evidence to demonstrate the survival of the clade into the Early Cretaceous. This includes a partial right ilium from Spain [63], a metacarpal from Bexhill, East Sussex [64], a skid-like chevron from the Isle of Wight [65], [66], and a caudal vertebra from China [34]. All these materials were the focus of discussion [12], [32], [34], and the conclusion is that none of them can be confidently attributed to a diplodocoid sauropod.

In this context, the discovery of *Leinkupal laticauda* in rocks belonging to the Early Cretaceous of South America represents not only the first certain diplodocid for any Cretaceous locality, but also the first for South America at any time. The new record demonstrates that although all diplodocoids originated during Middle Jurassic times, probably around Bajocian or Bathonian times, diplodocids thrived in Late Jurassic times and survived in southern continents until the Early Cretaceous. Dicraeosaurids thrived during Late Jurassic and Early Cretaceous times, and rebbachisaurids only thrived during the early Late Cretaceous, the former in western Laurasia and the latter on southern continents.

Carballido et al. [36] proposed that the main neosauropod clades should have originated only by the late Mid Jurassic, but because the Middle and Upper Jurassic was times of maximum marine transgressions[67], a passage for broad movements of terrestrial tetrapods only existed earlier or later. Though Remes [11] considered that most Gondwanan diplodocoids were less derived in morphology, suggesting a southern origin for the group, this is not true for any member of the flagellicaudatan clade. More accurately, all diplodocoid basal forms show a peculiar global distribution (as Remes considers as a second hypothetical option), suggesting high dispersive capabilities through wide distances and varied environments, including deserts. In this context, it is highly possible that rebbachisaurids never entered North America or became extinct prior to the depositation of the Morrison Formation, and dicraeosaurids never entered Europe. This is in agreement with observations of other widespread Mid-Jurassic to Early Cretaceous tetrapods with sister groups at any side of the desert that obviously were not severely affected by such environment, such as the ‘elaphrosaurs’, ceratosaurids, basal tetanurans [68], and basal eusauropods [69].

However, other groups seem to show different consequences concerning the existence of physical barriers. A recent DEC (Dispersal Extinction Cladogenesis) study of the well-known Cañadón Asfalto Basin [70] for ancestral reconstructions demonstrated the existence of remarkable southern provincialism in 11 out of 15 taxa. Though the early regionalization of Gondwana was already explored by Bonaparte [38] and the environmental causes (Central Gondwanan Desert; magmatic fields) acting as barriers were observed by several authors [57], [71], [69], discoveries during the last decade [72] permitted the addition of abundant fossil evidence to support the hypothesis that the differentiation was at least present since late Early Jurassic times, before the effective Pangean breakup, supporting the action of environmental barriers. This is probably related to a preference for the wet-dry biome marked by Jurassic plant groups with a Pangean distribution but closest peri-Antarctic affinities [72]. Furthermore, several originally Pangean groups radiated in Gondwanan localities after the opening of the Hispanic corridor, including cypresses [72], heterodontosaurid ornithischians [73], basal sphenodontids [74], local dryolestoids, gondwanatheres, some triconodont groups (e.g., *Condorodon* - *Tendagurodon*), but not others (*Volaticotherium – Argentoconodon*) [75], and Henosferidae mammals [76], to cite a few.

The phylogenetic position of *Leinkupal* situates this form not only within Diplodocidae but among diplodocines, a clade that excludes *Apatosaurus*. In this context, the diplodocines must have radiated worldwide no later than the Middle Jurassic, pushing the origin of Diplodocoidea to an early point during the Middle Jurassic or even earlier.

On the other hand, a late arrival of diplodocids to South America could be only explained by its presence in Africa since early to mid-Jurassic times, and no later, when all continental passes were shut. Though a land corridor was available from North America and Iberia by Late Jurassic times, the connection to Africa was via Apulia [55], [53]. This suggests the early dispersal model as the only way to explain this paleobiogeographic scenario.

## Conclusions

Though represented by fragmentary material, *Leinkupal laticauda* from Early Cretaceous strata of Argentina suggests that the supposed extinction of the Diplodocidae at the Jurassic/Cretaceous boundary didn't occur globally, but that the clade survived in South America at least during part of the Early Cretaceous. This was alongside the other two major diplodocoid clades, plus abundant macronarian clades, an extremely rich association not recorded before. The analysis of the Bajada Colorada fauna shows that diplodocoids were diverse and abundant in the Lower Cretaceous of Patagonia. Whereas flagellicaudatans are recorded in the Early Cretaceous, their sister-group, the Rebbachisauridae, succeeded during the early Late Cretaceous, after the extinction of the former, suggesting some degree of ecological replacement.

The phylogenetic analysis suggests a closer relationship of *Leinkupal* to the African *Tornieria*, showing a widespread and early distribution of diplodocids in South America or, alternatively, a colonization of South America from Africa by the Jurassic/Cretaceous boundary. The position of *Leinkupal* as a derived diplodocine sauropod pushes the origin of Diplodocoidea to the early Middle Jurassic or potentially even earlier.

Diplodocids are the most emblematic sauropod dinosaurs. The recognition of a member of this clade in Argentina augments the list of known sauropod clades for this country, thus representing an extremely rich portrait of sauropod evolution.

## Supporting Information

Figure S1 **Images of the fossil quarry where the remains assigned** ***Leinkupal laticauda*** **were recovered.**
(TIF)

Table S1 Vertebral measurements of *Leinkupal laticauda.*
(DOC)

Text S1 **Information concerning terminal taxa, character list and data matrix used in phylogenetic analysis.**
(DOC)

Text S2 **List of synapomorphies.**
(DOC)

Dataset S1 **Data matrix 1 (Modified from** **[10]).**
(NEX)

Dataset S2 **Data matrix 2 (Modified from** **[21]).**
(NEX)
