# Supplementary figures and images for: A Diplodocid Sauropod Survivor from the Early Cretaceous of South America
Source: PLoS One. 2014 May 14;9(5):e97128. doi: 10.1371/journal.pone.0097128 (PMC4020797; doi:10.1371/journal.pone.0097128)

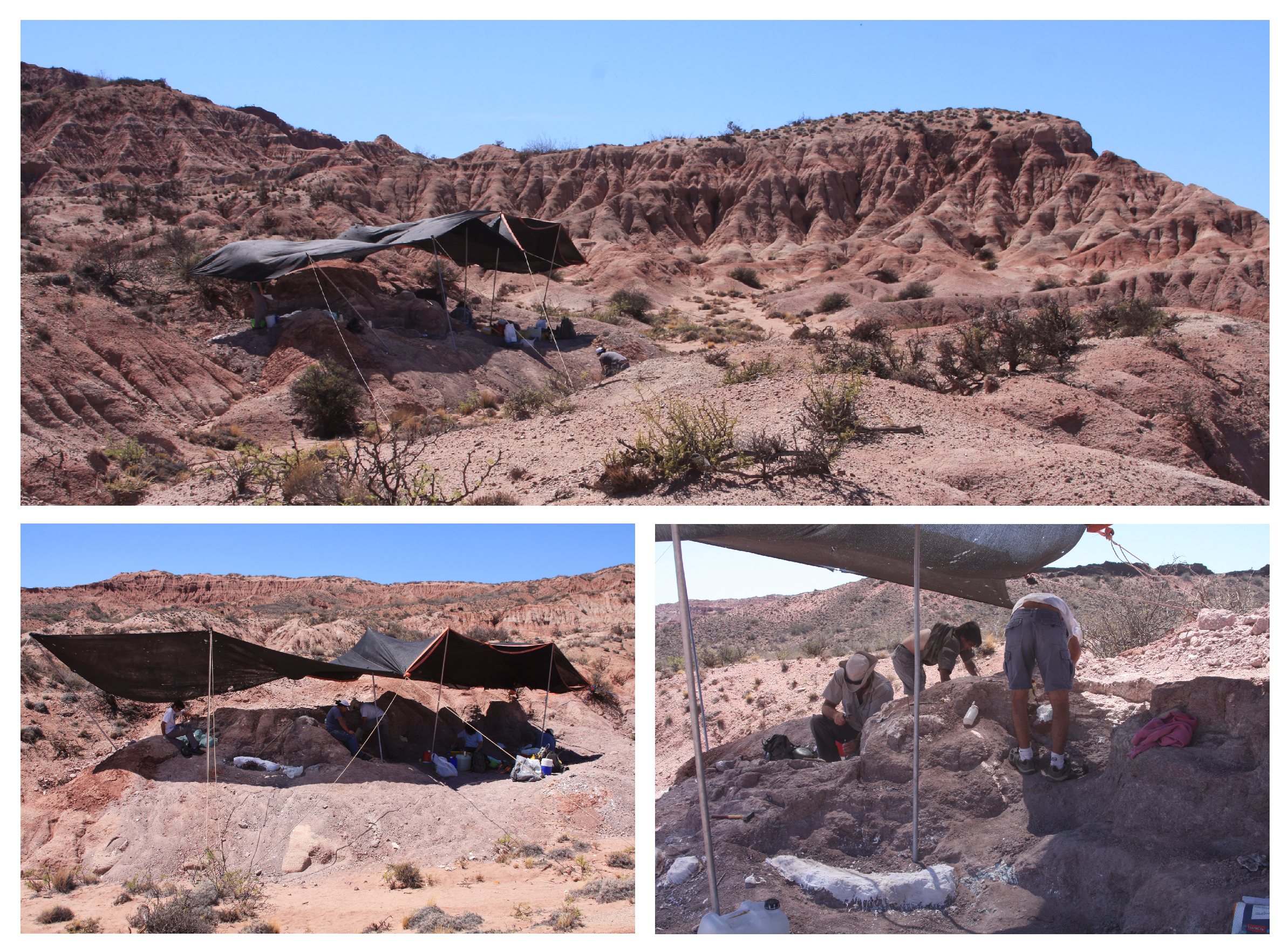

Supplement: Figure S1 — Images of the fossil quarry where the remains assigned Leinkupal laticauda were recovered. (TIF) [file pone.0097128.s001.tif]
