# Supplementary material for: A Diplodocid Sauropod Survivor from the Early Cretaceous of South America
Source: PLoS One. 2014 May 14;9(5):e97128. doi: 10.1371/journal.pone.0097128 (PMC4020797; doi:10.1371/journal.pone.0097128)
Supplement: Table S1 — Vertebral measurements of Leinkupal laticauda. (DOC) [file pone.0097128.s002.doc]

Table S1. Vertebral measurements (cm) of *Leinkupal laticauda*

|  |  |  |  | Preserved measurements | |  |
| --- | --- | --- | --- | --- | --- | --- |
| Element | Centrum Length | Centrum Width | Centrum Height | Total Length | Total Width | Total Height |
| C6 | 14.5 | 5* | 6.7 | 16.3 | 6.5 | 12 |
| C8 | 20.3 | 7.4* | 8.5* | 20.3 | 9.5 | 21 |
| C11 | 19.6 | 7* | 9.3 | 21 | 7 | 22.5 |
| D2 | 14 | 7.4* | 10.5 | 17.5 | 9.5 | 26.5 |
| Ca1-2 | 6 | 16 | 15 | 6 | 35.5 | 29 |
| Ca7 | 8.5 | 15 | 11.5 | 13 | 43 | 33 |
| Ca12 | 9 | 10.5 | 10 | 16 | 11.5 | 22 |
| Ca20 | 11 | 8.6 | 9 | 14.5 | 8.6 | 16 |

Estimated measurements are marked with asterisk.
