# Supplementary material for: A Diplodocid Sauropod Survivor from the Early Cretaceous of South America
Source: PLoS One. 2014 May 14;9(5):e97128. doi: 10.1371/journal.pone.0097128 (PMC4020797; doi:10.1371/journal.pone.0097128)
Supplement: Text S1 — Information concerning terminal taxa, character list and data matrix used in phylogenetic analysis. (DOC) [file pone.0097128.s003.doc]

TEXT S1. INFORMATION CONCERNING TERMINAL TAXA, CHARACTER LIST AND DATA MATRIX USED IN PHYLOGENETIC ANALYSIS.

Two analyses were carried out with different terminal taxa, character list and data matrix. First analysis includes the same terminal taxa, character list and data matrix of [1] with the addition of two new character (Characters 199 and 200) (see Dataset S1). The second analysis includes the terminal taxa, character list and data matrix of [2] with the addition of the same two new character (Characters 235 and 236) and the exclusion of *Amphicoelias* as terminal taxon because of its fragmentary nature (see Dataset S2). All multi−state characters were treated as unordered.

The new characters of both analyses are listed below.

**New characters**

(199) (235) Anterior caudal transverse processes (excluding the first) with distal tip ventrally projected; present (1), absent (0).

(200) (236) Mid-caudal vertebrae with lateral pneumatic fossae; present (1), absent (0).

**References (Text S1)**

1. Mannion PD, Upchurch P, Mateus O, Barnes R, Jones MEH (2012) New information on the anatomy and systematic position of *Dinheirosaurus lourinhanensis* (Sauropoda: Diplodocoidea) from the Late Jurassic of Portugal, with a review of European diplodocoids. Journal of Systematic Palaeontology 10: 521–551.

2. Tschopp E, Mateus O (2012) The skull and neck of a new flagellicaudatan sauropod from the Morrison Formation and its implication for the evolution and ontogeny of diplodocid dinosaurs. Journal of Systematic Palaeontology 11: 853–888.
