# Supplementary material for: A Diplodocid Sauropod Survivor from the Early Cretaceous of South America
Source: PLoS One. 2014 May 14;9(5):e97128. doi: 10.1371/journal.pone.0097128 (PMC4020797; doi:10.1371/journal.pone.0097128)
Supplement: Text S2 — List of synapomorphies. (DOC) [file pone.0097128.s004.doc]

**TEXT S2. LIST OF SYNAPOMORPHIES.**

**Analysis 1**

Diplodocoidea

Char. 132

Rebbachisauridae

Char. 142

Char. 154

Flagellicaudata

Char. 7

Char. 36

Char. 55

Char. 117

Char. 121

Char. 129

Char. 178

Char. 181

Char. 182

Char. 185

Char. 196

Dicraeosauridae

Char. 2

Char. 35

Char. 38

Char. 42

Char. 44

Char. 57

Char. 88

Char. 102

Char. 105

Char. 137

Char. 153

Diplodocidae

Char. 78

Char. 132

Diplodocinae

Char. 76

Char. 85

Char. 151

**Analysis 2**

Diplodocoidea

Char. 14

Char. 32

Char. 57

Char. 68

Char. 78

Char. 81

Char. 135

Char. 142

Char. 144

Char. 155

Char. 164

Char. 166

Char. 179

Char. 182

Char. 187

Char. 204

Char. 222

Char. 229

Flagellicaudata

Char. 39

Char. 41

Char. 97

Char. 102

Char. 154

Char. 162

Char. 170

Char. 194

Char. 210

Char. 213

Char. 217

Dicraeosauridae

Char. 2

Char. 40

Char. 45

Char. 50

Char. 52

Char. 71

Char. 72

Char. 84

Char. 107

Char. 132

Char. 171

Diplodocidae

Char. 114

Char. 183

Char. 226

Char. 235

Diplodocinae

Char. 65

Char. 97

Char. 102

Char. 103

Char. 113

Char. 173

Char. 185

Char. 223

*Leinkupal* + *Torneria*

Char. 94
